# Supplementary material for: Naïve Human Macrophages Are Refractory to SARS-CoV-2 Infection and Exhibit a Modest Inflammatory Response Early in Infection
Source: Viruses. 2022 Feb 21;14(2):441. doi: 10.3390/v14020441 (PMC8875879; doi:10.3390/v14020441)

Supplementary material

Figure S1: **Differential cytokine/chemokine production levels modulated by SARS-CoV-2 in MDM.** Human XL Cytokine array of secreted factors in the cell supernatant of (**A**) mock-infected, (**B**) 24 hours post LPS stimulation, (**C**) 6 hpi (MOI:1) and (**D**) 24 hpi (MOI:1) MDM.

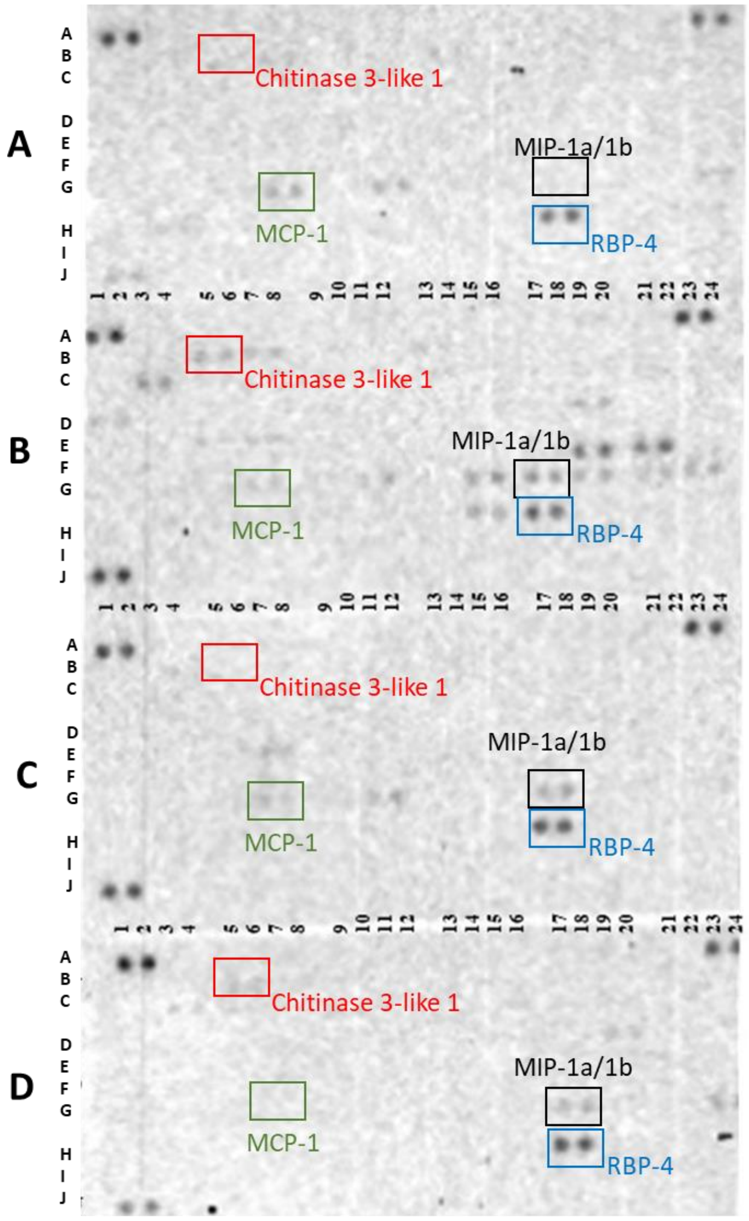

Figure S2: Replication of 6:2 Eng/09 and 6:2 Tky/05 influenza strains in MDM is abortive. Quantification of *M* gene expression levels in cell lysates (red) and supernatants (blue) of MDM infected with recombinant 6:2 Eng/09 (**A**) or 6:2 Tky/05 (**B**) influenza strains (MOI:1) at 6 and 24 hpi by qRT-PCR. Bars represent Mean ± SD.

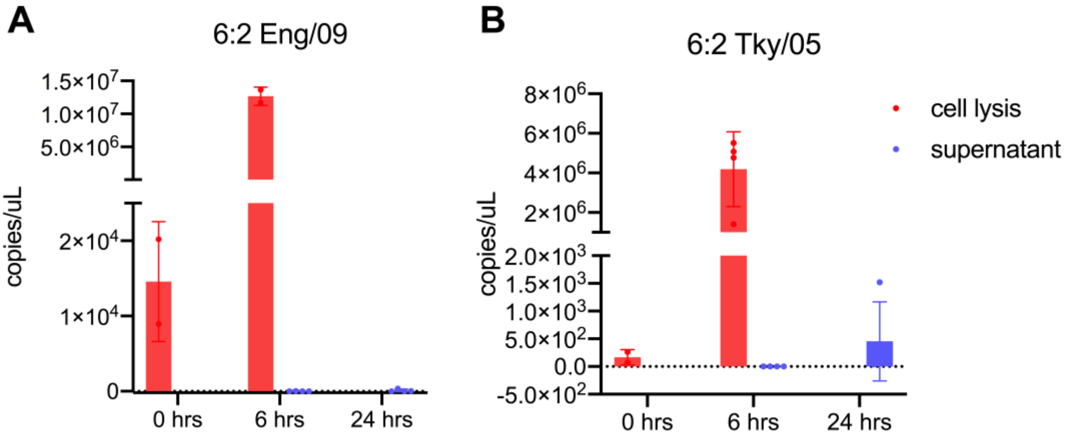

Supplement: Supplementary file 1 [file viruses-14-00441-s001.zip › viruses-1603793-supplementary.pdf]
